# Aortic length measurements for pulse wave velocity calculation: manual 2D vs automated 3D centreline extraction

**DOI:** 10.1186/s12968-017-0341-y

**Published:** 2017-03-08

**Authors:** Arna van Engelen, Miguel Silva Vieira, Isma Rafiq, Marina Cecelja, Torben Schneider, Hubrecht de Bliek, C. Alberto Figueroa, Tarique Hussain, Rene M. Botnar, Jordi Alastruey

**Affiliations:** 1 Department of Biomedical Engineering, Division of Imaging Sciences and Biomedical Engineering, King’s College London, St Thomas’ Hospital, 4th floor Lambeth Wing, Westminster Bridge Road, London, SE17EH UK; 2 Department of Cardiovascular Imaging, Division of Imaging Sciences and Biomedical Engineering, King’s College London, St Thomas’ Hospital, 4th floor Lambeth Wing, Westminster Bridge Road, London, SE17EH UK; 3 Department of Clinical Pharmacology, St Thomas’ Hospital, Westminster Bridge Road, London, SE17EH UK; 4 Philips Healthcare, Guildford, UK; 5 HSDP Clinical Platforms, Philips HealthTech, Best, The Netherlands; 6 Department of Bioengineering and Surgery, University of Michigan, Ann Arbor, MI USA; 7 Department of Pediatrics, Pediatric Cardiology, UT Southwestern Medical Center, Dallas, USA; 8 Pontificia Universidad Católica de Chile, Escuela de Ingeniería, Santiago, Chile

**Keywords:** Pulse wave velocity, Aortic stiffness, Centreline, Semi-automated tracking, Cardiovascular magnetic resonance

## Abstract

**Background:**

Pulse wave velocity (PWV) is a biomarker for the intrinsic stiffness of the aortic wall, and has been shown to be predictive for cardiovascular events. It can be assessed using cardiovascular magnetic resonance (CMR) from the delay between phase-contrast flow waveforms at two or more locations in the aorta, and the distance on CMR images between those locations. This study aimed to investigate the impact of different distance measurement methods on PWV. We present and evaluate an algorithm for automated centreline tracking in 3D images, and compare PWV calculations using distances derived from 3D images to those obtained from a conventional 2D oblique-sagittal image of the aorta.

**Methods:**

We included 35 patients from a twin cohort, and 20 post-coarctation repair patients. Phase-contrast flow was acquired in the ascending, descending and diaphragmatic aorta. A 3D centreline tracking algorithm is presented and evaluated on a subset of 30 subjects, on three CMR sequences: balanced steady-state free precession (SSFP), black-blood double inversion recovery turbo spin echo, and contrast-enhanced CMR angiography. Aortic lengths are subsequently compared between measurements from a 2D oblique-sagittal plane, and a 3D geometry.

**Results:**

The error in length of automated 3D centreline tracking compared with manual annotations ranged from 2.4 [1.8-4.3] mm (mean [IQR], black-blood) to 6.4 [4.7-8.9] mm (SSFP). The impact on PWV was below 0.5m/s (<5%). Differences between 2D and 3D centreline length were significant for the majority of our experiments (*p* < 0.05). Individual differences in PWV were larger than 0.5m/s in 15% of all cases (thoracic aorta) and 37% when studying the aortic arch only. Finally, the difference between end-diastolic and end-systolic 2D centreline lengths was statistically significant (*p* < 0.01), but resulted in small differences in PWV (0.08 [0.04 - 0.10]m/s).

**Conclusions:**

Automatic aortic centreline tracking in three commonly used CMR sequences is possible with good accuracy. The 3D length obtained from such sequences can differ considerably from lengths obtained from a 2D oblique-sagittal plane, depending on aortic curvature, adequate planning of the oblique-sagittal plane, and patient motion between acquisitions. For accurate PWV measurements we recommend using 3D centrelines.

## Background

Increased arterial stiffness is associated with vascular ageing and is an early predictor of cardiovascular risk [1–3]. Non-invasive surrogate measures of arterial stiffness include pulse pressure, distensibility, and pulse wave velocity (PWV). Of those, PWV is considered the ‘gold standard’ method to non-invasively quantify central aortic stiffness [1, 4]. In brief, in each cardiac cycle a pulse wave is generated by cardiac contraction and travels through the arterial vasculature with a certain velocity, known as PWV, which increases with arterial stiffening. Aortic PWV has been shown to be an independent predictor of cardiovascular events and all-cause mortality [3, 5]. Traditionally aortic PWV is assessed as carotid to femoral PWV, by determining the transit time between two pulse pressure or flow waveforms measured at the common carotid and right femoral artery, divided by an approximation of the travelled distance [1]. PWV measured as such has shown to be strongly correlated with age and blood pressure [6].

Cardiovascular magnetic resonance (CMR) enables for localised assessment of aortic PWV [7]. Studies have shown differences in PWV between the thoracic and abdominal aorta in normal subjects [8, 9], and found local differences in PWV in patients with abdominal aortic aneurysms [10] and Marfan’s disease [11, 12]. The most common approach in CMR-based studies is to measure the transit time from the arrival time of a pulse wave in two or more arterial locations from 2D time-resolved velocity-encoded phase-contrast (PC) CMR [13]. Previous studies have investigated different methods to obtain the transit time between two waveforms [14–16]. However, accurate estimation of the travel distance between waveform locations is equally important [4]. A common approach for CMR-based aortic PWV calculation is to use a 2D sagittal view of the aorta, either by directly obtaining these images [8–10, 17–21] or by using a reformatted oblique sagittal plane or MIP of a 3D volumetric acquisition [11, 12, 22–24]. Measuring the 3D vessel lengths may be more accurate due to the effects of out-of-plane curvature, however, longer 3D volumetric acquisitions are required. Wentland et al. showed differences between the described approach from 2D PC CMR and 4D flow CMR for which a 3D centreline was obtained. However, their work focused on the effect on the transit time using different temporal resolutions and did not analyse the critical contribution of differences in vessel length measurements [24].

Manual annotation of 3D centrelines can be challenging and time-consuming due to the need to inspect the centreline in three dimensions. Automated 3D centreline extraction methods would streamline PWV analysis, and possibly reduce the inter- and intra-observer variability. Automated aortic centreline tracking has been evaluated both on CT angiography [25–28] and CMR [29–31]. Often an initial lumen segmentation is obtained first, from which the centreline is extracted [26, 30, 31]. However, the segmentation process is time-consuming and potentially error-prone [32]. In other methods the centreline is directly extracted from the image itself [27–29]. Approaches include finding the centreline using image intensity in combination with an aortic model [29] and interactive circle-fitting along the artery [28]. In other cases such methods are often combined with a ‘vesselness’ filter [33], which, when applied to a 2D or 3D image, enhances vessel structures while reducing background signal. This filter is based on the Hessian matrix of the image, and has been used for automated analysis of a large variety of vessels [27, 34–37]. This approach was used by Krissian et al. [27], who identified a set of potential centrelines using the vesselness filter, and then manually selected the best one. An intrinsic factor of automatic algorithms is that their performance is optimised for certain imaging data and often needs to be modified for different MR contrast types.

This study aims to investigate different methodologies for aortic centreline measurements for PWV analysis. This paper consists of two parts: first, we propose and evaluate a 3D centreline tracking algorithm on three of the most commonly used CMR sequences. Second, we apply this algorithm to a larger dataset to evaluate the difference between 2D and 3D centreline length measurements in the aorta and the impact of these differences on PWV measurements. We used two different cohorts of patients: a group of healthy ageing twins, and a group of post-coarctation repair patients who often have altered aortic geometries.

## Methods

**Fig. 1:**
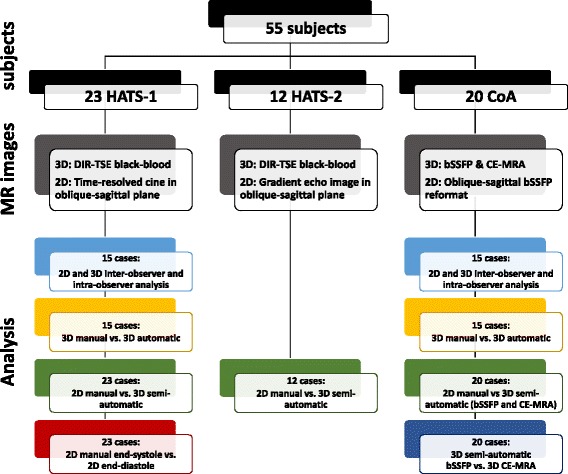
Overview of the included subjects, the acquired images and performed analyses. HATS = Healthy Ageing Twin Study, CoA = Coarctation study, DIR-TSE = double inversion recovery turbo spin echo, bSSFP = balanced steady-state free precession, CE-MRA = contrast-enhanced magnetic resonance angiography

A schematic overview of the patient data, CMR sequences, and performed analyses is provided in Fig. 1. In this study, we will obtain centrelines from both 3D and 2D CMR images. A centreline is a series of points in 3D space located in the centroid of a vessel. Here, we will also use the term centreline to refer to the points located on the centroid of a vessel on a 2D image.

### Imaging data

Data of 55 subjects were retrospectively selected from two cohorts: 35 subjects from the Healthy Ageing Twin Study (HATS) as part of the TwinsUK Registry (all female, age 69 ± 7 years) [38] and 20 subjects from a cohort of patients with non-stented surgically repaired aortic coarctation (CoA, 13 male, age 27 ± 8 years).

**Fig. 2:**
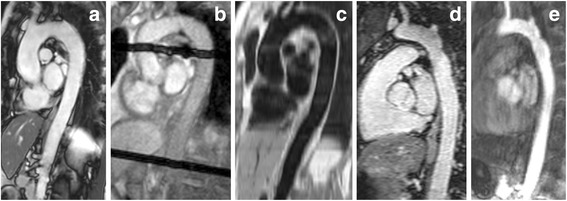
Examples of images used. For 2D centreline analysis: (**a**) oblique-sagittal slice of a 2D cine, and (**b**) oblique-sagittal GRE image with transverse saturation slabs indicating the positions of the PC-CMR images. For 3D centreline analysis: oblique-sagittal reformat from volumetric (**c**) DIR-TSE Black-Blood, (**d**) bSSFP and (**e**) contrast-enhanced MRA

CMR images were acquired on a 1.5T Philips Ingenia (HATS-1 and CoA) or Achieva (HATS-2) scanner (both Philips Healthcare, Best, the Netherlands). Sequence details are provided in Table 1 and Fig. 2 shows examples of the acquired images. For all patients free-breathing high-temporal resolution 2D through-plane velocity-encoded PC-CMR in the ascending (ASC), descending (DESC) and diaphragmatic (DIAPH) aorta were obtained.

**Table 1:** CMR scan protocol

	PC-CMR	2D cine	2D GRE	DIR-TSE	DIR-TSE	3D bSSFP	CE-MRA
Cohort	all	HATS-1	HATS-2	HATS-1	HATS-2	CoA	CoA
TE (ms)	2.7 ± 0.2	1.8 ± 0.3	1.3	13.4 ± 0.5	5.0	1.5 ± 0.2	1.1 ± 0.1
TR (ms)	4.5 ± 0.4	3.7 ± 0.6	4	1684 ± 242	1330 ± 466	3.6 ± 0.2	3.8 ± 0.1
Acquisition	Oblique-sagittal, single-slice	Oblique-sagittal, single-slice	Oblique-sagittal, single-slice	Axial, multi-slice	Axial, multi-slice	Coronal, 3D volumetric acquisition	Coronal, 3D volumetric acquisition
Acquired resolution (mm)	2.2 ± 0.1	1.4 ± 0.4	2.0	1.5 x 1.9 ± 0.1	1.0	1.4 ± 0.2	1.8
Reconstructed in-plane voxel size (mm)	1.1 ± 0.1	0.9 ± 0.3	1.8	1.1 ± 0.04	0.3	0.8 ± 0.2	1.2 ± 0.01
Slice thickness (mm)	8–10	8–10	15	5	5	1.6 ± 0.7	1.8
Temporal resolution (ms)	8.4 ± 5.5	24.5 ± 4.7	-	-	-	-	-
FA (°)	15–20	45–60	30	90	90	70	30
SENSE factor	1	1	1	1	1	1.2 ± 0.3	1.5

*TE* echo time, *TR* repetition time, *FA* flip angle

Due to the retrospective nature of the study, the data consisted of different CMR sequences. The 3D centreline tracking method was evaluated in 1) multi-slice 2D double inversion recovery turbo spin echo (DIR-TSE) black-blood images for the HATS cohort, and 2) 3D balanced steady-state free precession (bSSFP) and 3D contrast-enhanced MR Angiography (CE-MRA; 0.2mmol/Kg of *Gadovist* ^®^; *Bayer Schering Pharma*; Berlin, Germany) for the CoA patients.

For measuring the 2D lengths two different imaging sequences had been acquired in different subjects in the HATS cohort: 1) an oblique-sagittal 2D cine of the aorta for 23 of the HATS subjects (called HATS-1 hereafter) and 2) a single-slice oblique-sagittal gradient echo (GRE) image with transverse saturation slabs applied to indicate the positions of the PC-CMR images [10] for the remaining 12 subjects (HATS-2). For the CoA patients a third approach was used, which was to obtain an oblique-sagittal plane by reformatting the 3D bSSFP image.

The different sequences used in this study were acquired as part of a longer imaging protocol, with a total imaging time of about 1 h.

### Manual centreline annotations

Manual annotations of the aortic path were made on 3D images for evaluation of the automated centreline tracking algorithm, as well as on 2D images to compare 2D-derived length with the corresponding 3D-derived length.

**Fig. 3:**
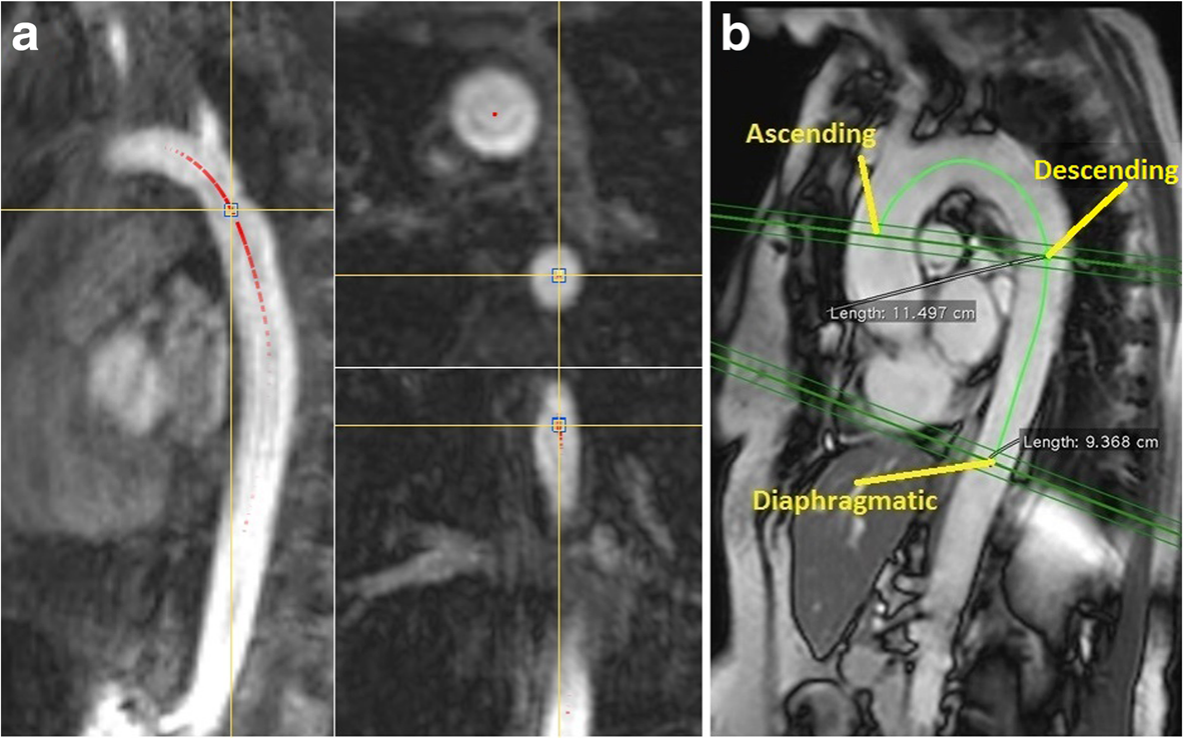
Manual annotation in (**a**) 3D and (**b**) 2D viewer

The 3D centreline tracking was first evaluated on 30 subjects: 15 randomly selected HATS-1 and 15 randomly selected CoA patients. For those 30 subjects manual annotation on both bSSFP and CE-MRA images was performed using a custom-made tool in MeVisLab (V2.6.1), which allowed annotation and inspection of the centrelines in the axial, coronal and sagittal imaging planes simultaneously (Fig. 3a). The manual centrelines were cut at the point closest to the centre of the cross-sectional lumen of the ASC, DESC and DIAPH aorta in the PC-CMR images.

The 2D annotations for the arch and thoracic aorta were manually obtained on all datasets using OsiriX (V.7.5). Start and end points for manual annotation were defined by projection of the intersection of the PC-CMR flow planes (Fig. 3b). For all HATS-1 subjects the distances were measured both at end-systole and end-diastole.

Intra- and inter-observer variability was assessed for both 3D and 2D manual annotation. This was performed on the 30 subjects used for the evaluation of automatic centreline tracking. For the 3D annotation method, one observer (AvE) annotated the centreline three times, and a second observer once (MSV for HATS-1, IR for CoA). For the 2D annotation method, lengths were also assessed three times by one observer (MSV for HATS-1 and AvE for CoA), and once by another observer (AvE for HATS-1 and IR for CoA). For HATS-1 the intra- and inter-observer variability was assessed at the end-diastolic frames. For CoA the reformatting of a 2D oblique-sagittal plane was also repeated for each annotation, so was also part of the intra- and inter-observer analysis.

We tracked the time it takes for manual annotation of 2D centreline on 5 HATS-1 and 5 CoA patients, and 3D annotation on 5 HATS-1, 5 CoA bSSFP and 5 CoA CE-MRA images, for one experienced observer (AvE).

### Automatic centreline tracking

The algorithm here presented is for use on volumetric images; both multi-slice 2D acquisition and true 3D acquisition techniques can be used, and it does not matter whether images are acquired in axial, coronal or sagittal orientation.

Automatic centrelines were computed in three steps: 1) vesselness filter [33], 2) fast marching [39] and 3) centreline refinement. The vesselness filter is commonly used for vascular image processing [27, 34–37]. It enhances vessel-like structures in an image by combining the eigenvalues of the Hessian matrix, composed of local second-order derivatives of the image, to get a maximum response at tubular structures. The Hessian matrix is computed at several scales, depending on the size of the vessel of interest, and the maximum response over the different scales is then taken at each voxel. More details can be found in [33]. We compared several scale settings for the Hessian matrix, based on the expected aortic diameters: using 4 scales, ranging from 4 to 7mm, and using 2 scales being either 4 and 6mm, or 6 and 8mm.

Bi-directional fast marching [39] was performed to find the most ideal path between a start and end point. Here a wavefront propagates from both ends using a speed map based on the vesselness. The start and end points for centreline tracking were defined by taking the centre of the ascending and diaphragmatic aorta on the first phase of the phase-contrast images. To account for patient movement in between the PC-CMR and the sequence used for centreline tracking, an ellipse was fitted on the artery in the 3D data [40, 41] at these points to re-center the start and end points. Finally, the centrelines were centred and smoothed by an open active contour [42]. The active contour makes use of two equally weighted forces: an internal force to minimise curvature and an external force to centre the contour.

This algorithm was implemented in a PWV prototype on the Philips IntelliSpace Discovery with clinical science extensions (Philips, Best, the Netherlands), working similarly to [43]. Manual adjustment of the obtained centreline was also possible in this prototype.

Automatic centreline tracking was evaluated on the 30 randomly selected patients for which manual annotations were made. To compare 3D and 2D distances, manual adjustment of the obtained 3D centrelines was performed to reposition control points in the centre of the vessel in cases where the algorithm produced inaccurate results. This tracking followed by manual correction was utilized in all subjects for the comparison between 2D and 3D centrelines. To avoid confusion, in this paper we refer to centrelines obtained using manual selection of the artery of interest on the PC-CMR without further manual interaction as automatic centrelines, and centrelines that have subsequently been adjusted as semi-automatic.

### Pulse wave velocity

Volumetric flow waveforms were obtained from the PC-CMR at the ascending, descending and diaphragmatic aorta, by fitting a circle to the vessel edge along a number of ray casts [40, 41] and propagating the segmentation to all phases [44]. The transit time describing the delay between the arrival of the pulse wave at two locations was subsequently computed using the foot-to-foot method [16]. The foot of each curve was determined based on the intersection of line tangent to the average maximum gradient during systole and a horizontal line through the local minimum. PWV was then calculated by dividing the centreline length between two locations by the transit time between those locations. For each subject, PWV calculations were performed for the segments ASC-DESC, DESC-DIAPH and for the entire thoracic aorta (ASC-DIAPH).

### Statistical analysis

Reproducibility and repeatability of 2D and 3D manual annotation were determined by looking at, respectively, inter- and intra-observer variation in centreline length. Subsequently, the centreline with median length of the observer who made 3 annotations was taken as the reference centreline for further analysis as described below.

For evaluation of the automatic centreline tracking in the 30 randomly selected subjects, the following steps were taken. First the number of failed tracings, defined as the centreline leaving the lumen, was counted. Then, the following parameters were obtained for non-failed cases: centreline length, the distance between manual and automatic centrelines, and PWV. All centrelines were resampled to a spacing of 0.1mm. Subsequently, the centrelines were split at the level of the descending aorta in the PC-CMR to obtain the ASC-DESC and DESC-DIAPH lengths separately. The minimum distance between manual and automatic centrelines was then calculated for each point along the resampled centrelines.

Lastly, a comparison between 2D and 3D aortic length, and corresponding PWV, was made. This analysis was performed for each individual dataset (HATS-1, HATS-2, CoA bSSFP and CoA CE-MRA). Results, separated for the different aortic segments, are presented using Bland-Altman analysis. Furthermore, for the 2D cine images in the HATS-1 subset, the difference between the end-diastolic and end-systolic length and resulting PWV was assessed. For the CoA cohort the difference between centrelines obtained from bSSFP and CE-MRA images was analysed. Statistical comparisons were made by a paired Wilcoxon signed ranks test due to non-normality of the underlying data as confirmed by a Kolmogorov-Smirnov test. As the DIR-TSE BB images were triggered at end-systole, the end-systolic 2D measurements were taken for the comparisons in the HATS-1 cohort.

We tested all results (length differences, PWV differences, centreline distances) for normality using a Kolmogorov-Smirnov test. Since the majority of results was not normally distributed, all results are presented with their median and interquartile range (IQR).

## Results

### Inter- and intra-observer variation

Inter- and intra-observer variation in centreline length for both 2D and 3D measurements are provided in Table 2. Centreline length annotation was generally more consistent for the HATS cohort than for the CoA patients. Additionally, both inter- and intra-observer variability was greater in the 2D measurement across all cohorts. However, absolute differences for both inter- and intra-observer assessments generally stayed well below 1 cm, or 5% of centreline length. Those differences were mostly caused by discrepancies in the aortic arch (ASC-DESC).

**Table 2:** Inter- and intra-observer variation in centreline length annotation (mm and %, provided as median [IQR])

		Absolute length difference (mm, %)		
		ASC-DESC	DESC-DIAPH	Total
HATS-1 2D (ED)	Intra-observer	2.3 [1.0–3.3], 1.8 [0.8–2.7]%	1.1 [0.4–1.6], 1.0 [0.3–1.5]%	2.6 [1.5–4.5], 1.1 [0.6–1.9]%
	Inter-observer	5.2 [3.4–7.9], 4.1 [2.7–5.8]%	0.7 [0.4–2.0], 0.7 [0.4–1.8]%	5.8 [3.1–8.0], 2.5 [1.5–3.5]%
CoA 2D	Intra-observer	2.7 [1.2–4.8], 2.4 [0.9–4.4]%	1.1 [0.5–1.7], 1.1 [0.4–1.7]%	2.8 [1.7–3.9], 1.2 [0.7–1.9]%
	Inter-observer	5.6 [3.7–7.7], 5.2 [3.4–6.9]%	2.1 [0.8–4.1], 1.6 [0.7–3.1]%	4.8 [2.8–6.9], 2.1 [1.2–3.2]%
HATS-1 3D	Intra-observer	0.9 [0.4–1.5], 0.8 [0.3–1.2]%	0.2 [0.1–0.4], 0.2 [0.1–0.3]%	0.9 [0.5–1.5], 0.4 [0.2–0.7]%
	Inter-observer	0.8 [0.4–2.1], 0.7 [0.3–1.7]%	0.4 [0.2–0.5], 0.4 [0.2–0.5]%	1.3 [0.5–2.5], 0.6 [0.2–1.2]%
bSSFP CoA 3D	Intra-observer	1.2 [0.5–2.1], 1.0 [0.5–1.7]%	0.2 [0.1–0.5], 0.2 [0.1–0.4]%	1.3 [0.6–2.5], 0.6 [0.3–1.0]%
	Inter-observer	2.3 [1.5–3.9], 2.0 [1.3–3.2]%	0.7 [0.3–0.9], 0.7 [0.2–1.1]%	2.8 [1.6–4.7], 1.4 [0.7–1.8]%
CE-MRA CoA 3D	Intra-observer	0.9 [0.3–1.7], 0.8 [0.3–1.8]%	0.3 [0.1–0.7], 0.3 [0.1–0.6]%	1.0 [0.5–1.9], 0.4 [0.2–0.7]%
	Inter-observer	2.9 [1.7–5.6], 2.5 [1.5–4.6]%	0.8 [0.3–1.5], 0.6 [0.2–1.2]%	3.0 [0.8–6.5], 1.4 [0.4–2.8]%

Pure annotation time in 2D did not differ much between HATS-1 and CoA annotation. Setting the start and end points took on average 21.1s, tracking ASC-DESC 15.3s, and tracking DESC-DIAPH 12.9s, with a total average of 49.3s (range 42.6–58.1s). For the CoA patients additionally time for reformatting the bSSFP image was on average 22.3s (range 13.2–31.4). Annotation of a 3D centreline took 2.11 min for CoA CE-MRA, 2.24 min for CoA bSSFP and 1.12 min for HATS-1 black-blood DIR TSE. These centrelines were automatically cut at the start and end points, and split in two afterwards.

### Automatic centreline tracking

**Fig. 4:**
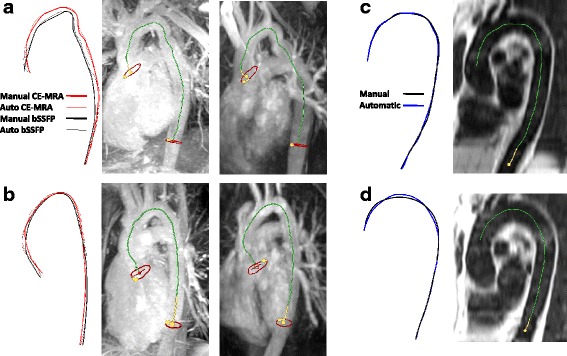
Automatic tracking results. **a**, **b** CoA patients with the automatic result shown on a volumetric maximum intensity projection of bSSFP (left) and CE-CMR (right), (**c**, **d**) results for HATS patients with the obtained centerline projected on a sagittal plane

The tracking results for the automatic centreline method using different scale settings are presented in Table 3. A few examples of obtained centrelines are shown in Fig. 4.

**Table 3:** Results for automatic centreline tracking vs. manual annotation: length differences, point-based centreline distances, and corresponding PWV accuracy, all provided as median [IQR]

	Failed tracings	Absolute length difference (mm)	Average centreline distance (mm)	Absolute PWV difference (m/s + %)
HATS-1				
Scales: 4, 5, 6, 7 mm	-	4.0 [1.9–5.0]	1.3 [0.9–2.0]	0.13 [0.08–0.21], 1.9 [0.9–2.0]%
Scales: 4, 6 mm	-	2.4 [1.8–4.3]	1.3 [0.8–1.9]	0.08 [0.06–0.19], 1.1 [0.8–1.8]%
Scales: 6, 8 mm	-	5.4 [3.7–8.0]	1.5 [1.0–2.3]	0.22 [0.16–0.28], 2.4 [1.7–3.3]%
CoA bSSFP^a^				
Scales: 4, 5, 6, 7 mm	5	7.3 [5.6–8.2]	1.6 [1.0–2.7]	0.15 [0.11–0.20], 2.9 [2.5–3.2]%
Scales: 4, 6 mm	3	6.4 [4.7–8.9]	1.5 [0.9–2.7]	0.15 [0.09–0.21], 2.8 [2.0–4.7]%
Scales: 6, 8 mm	5	8.0 [7.1–9.9]	2.0 [1.2–4.1]	0.16 [0.12–0.21], 3.1 [2.6–4.0]%
CoA CE-MRA				
Scales: 4, 5, 6, 7 mm	-	3.9 [2.7–6.3]	1.2 [0.8–1.9]	0.09 [0.05–0.13], 1.5 [1.2–2.8]%
Scales: 4, 6 mm	-	2.9 [1.9–4.9]	1.2 [0.7–1.9]	0.07 [0.03–0.12], 1.5 [0.8–2.6]%
Scales: 6, 8 mm	-	5.0 [3.5–8.1]	1.4 [0.9–2.3]	0.11 [0.08–0.18], 2.3 [1.5–3.6]%

^a^Results for bSSFP are after excluding failed centrelines

The method only failed in a fraction of the bSSFP images and produced valid results in all other image types. Overall, out of the three different methods for calculating the Hessian matrix, using 2 scales (4 and 6 mm) provided the best results. Relative to manual annotations this produced length differences below 1 cm, and corresponding PWV differences well below 0.5 m/s (both <5%), and the smallest rate of failure in the bSSFP images (3/15). The largest differences with manual annotation in centreline length were seen for the bSSFP images. A more detailed analysis differentiating between the ASC-DESC and DESC-DIAPH segments for tracking using the optimised settings is given in Table 4. Differences in length and PWV were larger for the ASC-DESC segment than for the DESC-DIAPH segment.

**Table 4:** Results for best chosen centreline algorithm (scale 4–6mm), split between the arch (ASC-DESC) and descending aorta (DESC-DIAPH)

	Absolute length difference (mm)		Average centreline distance (mm)		Absolute PWV difference (m/s and %)	
	Arch	DESC	Arch	DESC	Arch	DESC
HATS-1	2.7 [1.4–4.3]	0.2 [0.1–0.5]	1.7 [1.1–2.6]	1.1 [0.7–1.4]	0.21 [0.11–0.35], 2.6 [1.8–3.6]%	0.02 [0.01–0.05], 0.2 [0.1–0.4]%
CoA bSSFP^a^	4.8 [3.6–7.4]	1.5 [0.6–2.4]	2.0 [1.2–3.4]	1.3 [0.8–2.3]	0.26 [0.15–0.31], 4.2 [3.2–5.9]%	0.06 [0.02–0.09], 1.3 [0.4–1.9]%
CoA CE-MRA	2.4 [0.9–4.3]	0.5 [0.4–1.3]	1.3 [0.8–2.1]	1.2 [0.7–1.7]	0.12 [0.04–0.18], 2.3 [0.9–3.7]%	0.03 [0.01–0.05], 0.7 [0.3–1.0]%

^a^Results for bSSFP are after excluding failed centrelines

### Differences between approaches

**Fig. 5:**
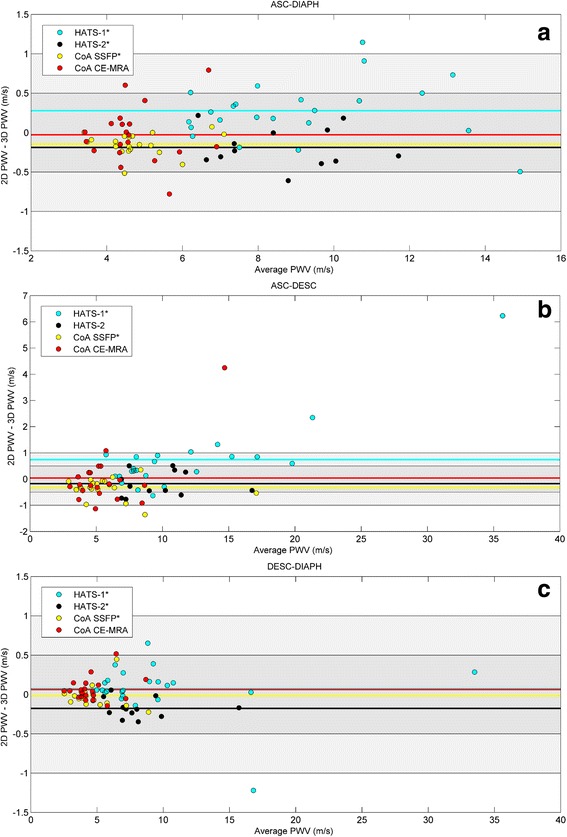
Bland-Altman plots depicting 2D PWV versus 3D PWV, for (**a**) ASC-DIAPH, (**b**) ASC-DESC and (**c**) the DESC-DIAPH segment. Shaded areas indicate the difference < 0.5 m/s and 1 m/s. Different cohorts are shown with different colors. The average difference for each cohort is indicated by the correspondingly colored line. For clarity of the figure the 95% confidence intervals are not shown

The comparison between 2D and 3D methods for length measurements for the full aorta segment (ASC-DIAPH) is provided in Table 5. The difference in PWV, specified per aortic segment, is also depicted in Bland-Altman plots in Fig. 5, with corresponding limits of agreement provided in Table 6.

**Table 5:** Comparison between different methods of measuring centreline length

	Difference length (mm)	Difference PWV (mean ± std, %)	Absolute Difference PWV (mean ± std, %)
2D–3D			
HATS-1**	7.4 [2.4–11.6]	0.26 [0.08–0.48], 3.0 [1.1–4.9]%	0.28 [0.17–0.50], 3.3 [2.3–4.9]%
HATS-2*	−6.9 [−8.8–0.3]	−0.26 [−0.35–0.02], −2.7 [−4.1–0.2]%	0.26 [0.16–0.35], 3.2 [1.8–4.1]%
CoA bSSFP**	−6.3 [-10.8 – −2.1]	−0.13 [−0.22 – −0.04], −3.1 [−4.5 – −1.0]%	0.13 [0.05–0.22], 3.1 [1.1–4.5]%
*CoA CE-MRA*	−4.0 [−13.5–6.5]	−0.07 [−0.24–0.11], −1.6 [−4.9–2.6]%	0.18 [0.11–0.38], 3.7 [2.5–7.5]%
ED-ES**	−1.5 [−3.2 – −1.3]	−0.08 [−0.10 – −0.04], −0.6 [−1.4 – −0.5]%	0.08 [0.04–0.10], 0.6 [0.5–1.4]%
bSSFP-CE-MRA	7.8 [−8.1–14.4]	0.14 [−0.13–0.25], 2.9 [−3.6–5.4]%	0.22 [0.13–0.30], 4.2 [3.4–6.7]%

2D manual minus 3D semi-automatic length, end-diastolic (ED) minus end-systolic (ES) length, and length from bSSFP minus CE-MRA (*= *p* ≤ 0.05, **= *p* ≤ 0.01, calculated for the PWV difference). ‘Difference length’ and ‘Difference PWV’ indicate whether a bias is present, whereas ‘absolute difference PWV’ indicates the average difference between the methods, disregarding a bias between the two. All results are provided as median [IQR]

**Table 6:** Average and limits of agreement for the PWV data presented in Fig. 5

	ASC-DIAPH	ASC-DESC	DESC-DIAPH
HATS-1	0.28 [−0.44 1.00]	0.74 [−1.91 3.40]	0.07 [−0.58 0.72]
HATS-2	−0.19 [−0.68 0.30]	−0.17 [−1.11 0.76]	−0.18 [−0.42 0.07]
CoA bSSFP	−0.15 [−0.42 0.13]	−0.33 [−1.09 0.43]	−0.02 [−0.29 0.26]
CoA CE-MRA	−0.03 [−0.72 0.67]	0.04 [−2.16 2.24]	0.06 [−0.23 0.35]

Differences can be seen between datasets, with significant differences for HATS-1 (2D > 3D), HATS-2 (3D > 2D) and the CoA bSSFP images (3D > 2D). The absolute difference between PWV derived from a 2D or a 3D centreline was above 0.5 m/s in 15% of our cases, and greater than 1 m/s in 1 case (1%). The limits of agreement were the smallest for the CoA bSSFP images.

Further sub-analysis of the absolute length difference between the 2D and 3D centreline for each of those patient groups, showed that significant differences were localised in the ASC-DESC segment. For ASC-DESC the overall absolute difference in PWV for all cohorts together was 0.38 [0.24–0.76] m/s (5.2 [3.1–9.9] %), and for DESC-DIAPH 0.09 [0.04–0.18] m/s (1.6 [0.7–2.8] %). Moreover, for the arch in 37% of cases the absolute difference in PWV was larger than 0.5 m/s, and in 11% larger than 1 m/s (with two outliers of 4.2 and 6.2 m/s, owing to both a very short transit time (4–5 ms) and a large length difference (2.3 and 2.6 cm)). For the descending segment a difference larger than 0.5 m/s was observed in only 4% of cases, and a difference larger than 1 m/s was found in 1% of all cases.

The difference between end-diastolic and end-systolic length measurements was −1.5 [−3.2 – −1.3] mm (ES > ED, *p* < 0.01). This discrepancy would lead to a difference in PWV estimation of 0.08 [0.04–0.10] m/s. Additionally, although the difference between the 3D centrelines measured on the bSSFP and CE-MRA images in the CoA cohort was not significantly different, the absolute differences in PWV were relatively large (4.2 [3.4–6.7]%, ranging up to 0.8 m/s).

## Discussion

We have shown that centrelines can be extracted accurately from 3D CMR images with minimal user interaction. Additionally, we have shown that obtaining the centreline from either a 2D or 3D anatomical image can result in significant differences in length, and therefore PWV.

In principle, the presented centreline tracking algorithm can be applied to any volumetric image, whether acquisition is 2D multi-slice or true 3D, and is independent of the orientation of the volume. The only requirement is a sufficiently high resolution and signal-to-noise ratio. Our results suggest that the image type, however, has an impact on the tracking performance. We obtained the most accurate centrelines on black-blood and contrast-enhanced images. The bSSFP images were more prone to failed tracking and showed larger differences in length. This can be explained by the larger intensity variations within the aorta, since signal loss is not uncommon in the presence of a high degree of turbulence or rapid jets across stenotic lesions. Moreover, this sequence was optimised as a cardiac sequence and not specifically for the aorta.

Three failures occurred on the bSSFP images using the optimal scale settings for the tracking algorithm. In one subject this was a small deviation outside of the lumen that could easily be adjusted manually by moving control points. In the other cases the centreline went through the pulmonary artery or the heart, due to signal dropout in the aortic arch. Besides manual correction, these errors could be overcome by adding one or more additional points in the lumen via which the tracking is performed.

The relatively small number of tracking failures on bSSFP, as well as the absence of any failed tracings on the DIR-TSE and CE-MRA highlights the robustness of the method with different imaging protocols, and demonstrates the potential for further evaluation or our proposed methodology in a practical clinical research workflow.

The intra- and inter-observer variation was larger for 2D analysis than for 3D. This highlights the importance of correct planning when 2D distance measurements are performed. Difficulties in accurate annotation arose mostly in cases where part of the aorta was not in the imaging plane, due to either aortic curvature or suboptimal planning. Additionally, start and end points were user defined on 2D images, while the annotated 3D centrelines were post-processed to start and end at automatically determined points. This makes it difficult to directly interpret the differences in 2D and 3D centreline length variability. Intra- and inter-observer variation was slightly larger for the CoA cohort, which can be explained by both the more complex geometry, and that for this dataset additional variability in the 2D analysis arises from selection of the oblique-sagittal plane. Even though requiring fewer steps (defining start, end, and position to split the centreline), manual annotation was faster on 2D images than on 3D images. For 2D annotation there was no difference between annotating HATS-1 and CoA datasets, but for 3D centrelines the CoA patients took twice as long, due to their more complex anatomy.

Centrelines obtained from a 2D image were expected to be shorter than with a 3D method, since out of plane curvatures are not captured with a 2D projection. This was indeed found to be the case for the HATS-2 dataset, where 2D distances were measured on a directly acquired 2D oblique-sagittal plane, and in the bSSFP images of the CoA cohort, where 2D distances were obtained from a plane obtained by reformatting a 3D image. In the latter case, we obtained 2D and 3D measurements from the same bSSFP images. This confirmed that by intersecting the aorta with one plane, shorter lengths are obtained (in 75% of cases) over the full aortic length. Nevertheless, we also found that the impact on estimated PWV was small with a difference in ASC-DIAPH PWV below 0.5 m/s in most cases. The limits of agreement on the Bland-Altman plot were smallest for the CoA bSSFP images. This is most likely because the 2D and 3D measurements were obtained from the same image, and variation is therefore only due to 2D/3D projection. For the other datasets, the 2D and 3D measurements were taken from different images leading to additional variations due to, for example, patient motion. Furthermore, the larger bias for datasets with higher PWV is in agreement with differences in centreline length having a larger effect on PWV in segments with shorter transit time, so stiffer arteries. Such a bias is not present when comparing 2D versus 3D centreline length.

Surprisingly, we found that for the HATS-1 cohort 3D distances were on average shorter than those obtained from 2D images. From a mathematical perspective, the projection of a 3D line onto a 2D plane cannot produce a longer length. After inspecting the cases with differences larger than 1 cm, we attributed this pattern to either patient motion or a suboptimal planning of the oblique-sagittal plane, which forced the observers to estimate the course of the aortic arch.

The difference between 2D and 3D centreline length and PWV was larger for the aortic arch than for the descending aorta. This is likely due to larger out-of-plane curvature in the arch. In addition, it should be noted that variations in segment length have a greater impact on PWV in shorter segments, and in cases with shorter transit times. Therefore, caution should be taken in the interpretation of PWV calculation performed with 2D measurements, especially in shorter or curved anatomies such as in the aortic arch.

As aortic length increases with age [28, 45], there might be a relationship between the difference between 2D and 3D length, and age. We did, however, not find such a correlation within any of the used datasets. This could be related with the small variation of age within each dataset.

The differences between bSSFP and CE-MRA 3D tracking was not significant, since one of the two was not consistently larger than the other. We therefore think differences are more likely due to patient motion between the scans than due to differences in the imaging protocol. This can for example be seen in Fig. 4a where a displacement of the arch is visible between the bSSFP and CE-MRA image. This results in different centreline lengths, especially in the arch, since the planes of the PC-CMR do not change position. This result implies that it is important to take patient motion into account when determining PWV. In order to minimise this effect, it is recommended to acquire the PC flow images and the image used for distance measurements close in time to each other.

4D PC-CMR could be used to overcome the problem of patient motion in between anatomical and flow scans. With this method, time-resolved velocity encoding in all three spatial directions is acquired with large volumetric coverage [24]. However, 4D PC-CMR is still limited by low temporal resolution, resulting in more difficult transit time assessment, and longer acquisition time.

In our results, PWV differed more than 0.5 m/s between using 2D or 3D centrelines in a considerable number of cases (15% full aorta, 37% arch). However, PWV is known to vary considerably between patients. The width of the IQR of aortic PWV in young healthy adults was shown to be about 1 m/s [21] using CMR. Furthermore, the carotid-femoral PWV in healthy adults (30–70 years old) was shown to vary within 3–5m/s (10^th^–90^th^ percentile) [46]. In this context, a difference of 0.5 m/s may not influence a clinical decision of diagnosis. Nevertheless, smaller differences as detected in our study can become relevant in the follow-up of individual patients with repetitive CMR scans, underlying the importance of measurement reproducibility.

The difference between end-systolic and end-diastolic aortic lengths was small (−1.5 [−3.2 – −1.3] mm), but significant. The longer distances for end-systolic measurements may be explained by aortic deformations during systole. As a result of aortic expansion, the centreline appears slightly higher in the axial direction along the arch. Although we did not have the data to confirm this using 3D images, given that the differences were so small we argue that the effect of measuring PWV either in end-systole or end-diastole can be neglected.

The main limitation of this study is the retrospective set-up. This caused different 2D and 3D images being available for the HATS and CoA cohorts, the absence of a single-slice oblique-sagittal 2D sequence for the CoA patients, and different acquisition settings for the DIR TSE sequence in the HATS-1 and HATS-2 population. However, we do argue that this set-up allowed us to study both centreline tracking and the effect of 2D versus 3D length measurements in different realistic clinical settings.

For both 2D and 3D centreline determination, the most important element of the MR image is that the aorta is clearly visible. Small differences in image quality, such as shown for the bSSFP images, may affect automatic 3D tracking. However, given an accurate centreline, possibly obtained after manual adjustment, the image type does not affect the PWV measurement. Acquisition aspects that do affect length measurements are aspects affecting positioning of the aorta, such as imaging at expiration or inspiration. The second part of this study, comparing 2D and 3D centreline geometries, showed that both projection to a 2D plane (shown on bSSFP images) and patient motion affect length measurements and therefore PWV.

Besides centreline length, transit time is the other important determinant in PWV analysis. In order to isolate this effect from the different approaches for distance measurement, transit time was maintained in each patient in this study. However, it is known that accurate transit time measurements are equally important as length measurements for accurate PWV calculation. Higher temporal resolutions and appropriate algorithms [16] can ensure more accurate transit time assessment. A previous study showed that estimates using the foot-to-foot method lead to relative errors in the range of 5–15%, thus having a larger effect on final PWV measurements [16].

## Conclusions

We have presented a new approach for obtaining accurate 3D centrelines from routine clinical CMR datasets with minimal user interaction. Moreover, we have shown significant differences between PWV calculated using centreline lengths obtained using a 3D or 2D method. Independent of the choice of distance measurement, patient motion was also shown to affect the PWV outcome. Although there are cases where the aortic geometry enables the acquisition of a well-planned oblique-sagittal plane suitable for accurate PWV measurements, special care should be taken when analysing short and/or tortuous segments such as the aortic arch. Because of these findings we recommend to calculate centreline length from a 3D image, and to acquire the images used to obtain transit time and vessel length consecutively, minimizing the chance of patient movement.

## Abbreviations

ASC: Ascending aorta
bSSFP: Balanced steady-state free precession
CE-MRA: Contrast-enhanced magnetic resonance angiography
CMR: Cardiovascular magnetic resonance
CoA: Coarctation dataset
DESC: Descending aorta
DIAPH: Diaphragmatic aorta
DIR-TSE: Double inversion-recovery turbo-spin echo
ED: End-diastolic
ES: End-systolic
FA: Flip angle
GRE: Gradient echo
HATS: Healthy ageing twins study
IQR: Inter-quartile range
PC: Phase-contrast
PWV: Pulse wave velocity
TE: Echo time
TR: Repetition time
